# Geospatial modelling of dry season habitats of the malaria vector, *Anopheles funestus*, in south-eastern Tanzania

**DOI:** 10.1186/s13071-024-06119-6

**Published:** 2024-01-29

**Authors:** Najat F. Kahamba, Fredros O. Okumu, Mohammed Jumanne, Khamisi Kifungo, Joel O. Odero, Francesco Baldini, Heather M. Ferguson, Luca Nelli

**Affiliations:** 1 Environmental Health and Ecological Sciences Department, Ifakara Health Institute, P. O. Box 53, Ifakara, Tanzania; 2 School of Biodiversity, One Health and Veterinary Medicine, University of Glasgow, Glasgow, UK; 3 School of Public Health, Faculty of Health Science, University of the Witwatersrand, Johannesburg, South Africa; 4 Wits Research Institute for Malaria, School of Pathology, Faculty of Health Sciences, University of the Witwatersrand, Johannesburg, South Africa; 5 School of Life Science and Biotechnology, Nelson Mandela African Institution of Science and Technology, P. O. Box 447, Arusha, Tanzania

**Keywords:** Habitat characterization, Larval ecology, Mosquito distribution, Environmental factors, Land cover analysis, Aquatic habitat mapping, Habitat suitability, Southeastern Tanzania

## Abstract

**Background:**

*Anopheles funestus* is a major malaria vector in Eastern and Southern Africa and is currently the dominant malaria-transmitting vector in many parts of Tanzania. Previous research has identified its preference for specific aquatic habitats, especially those that persist in dry months. This observation suggests the potential for targeted control through precise habitat mapping and characterization. In this study, we investigated the influence of habitat characteristics, land cover and human population densities on *An. funestus* distribution during dry seasons. Based on the results, we developed a habitat suitability model for this vector species in south-eastern Tanzania.

**Methods:**

Eighteen villages in south-eastern Tanzania were surveyed during the dry season from September-December 2021. Water bodies were systematically inspected for mosquito larvae and characterized by their physico-chemical characteristics and surrounding environmental features. A generalized linear model was used to assess the presence of *An. funestus* larvae as a function of the physico-chemical characteristics, land use and human population densities. The results obtained from this model were used to generate spatially explicit predictions of habitat suitability in the study districts.

**Results:**

Of the 1466 aquatic habitats surveyed, 440 were positive for *An. funestus*, with river streams having the highest positivity (74%; *n* = 322) followed by ground pools (15%; *n* = 67). The final model had an 83% accuracy in predicting positive *An. funestus* habitats, with the most important characteristics being permanent waters, clear waters with or without vegetation or movement and shading over the habitats. There was also a positive association of *An. funestus* presence with forested areas and a negative association with built-up areas. Human population densities had no influence on *An. funestus* distribution.

**Conclusions:**

The results of this study underscore the crucial role of both the specific habitat characteristics and key environmental factors, notably land cover, in the distribution of *An. funestus*. In this study area, *An. funestus* predominantly inhabits river streams and ground pools, with a preference for clear, perennial waters with shading. The strong positive association with more pristine environments with tree covers and the negative association with built-up areas underscore the importance of ecological transitions in vector distribution and malaria transmission risk. Such spatially explicit predictions could enable more precise interventions, particularly larval source management, to accelerate malaria control.

**Graphical Abstract:**

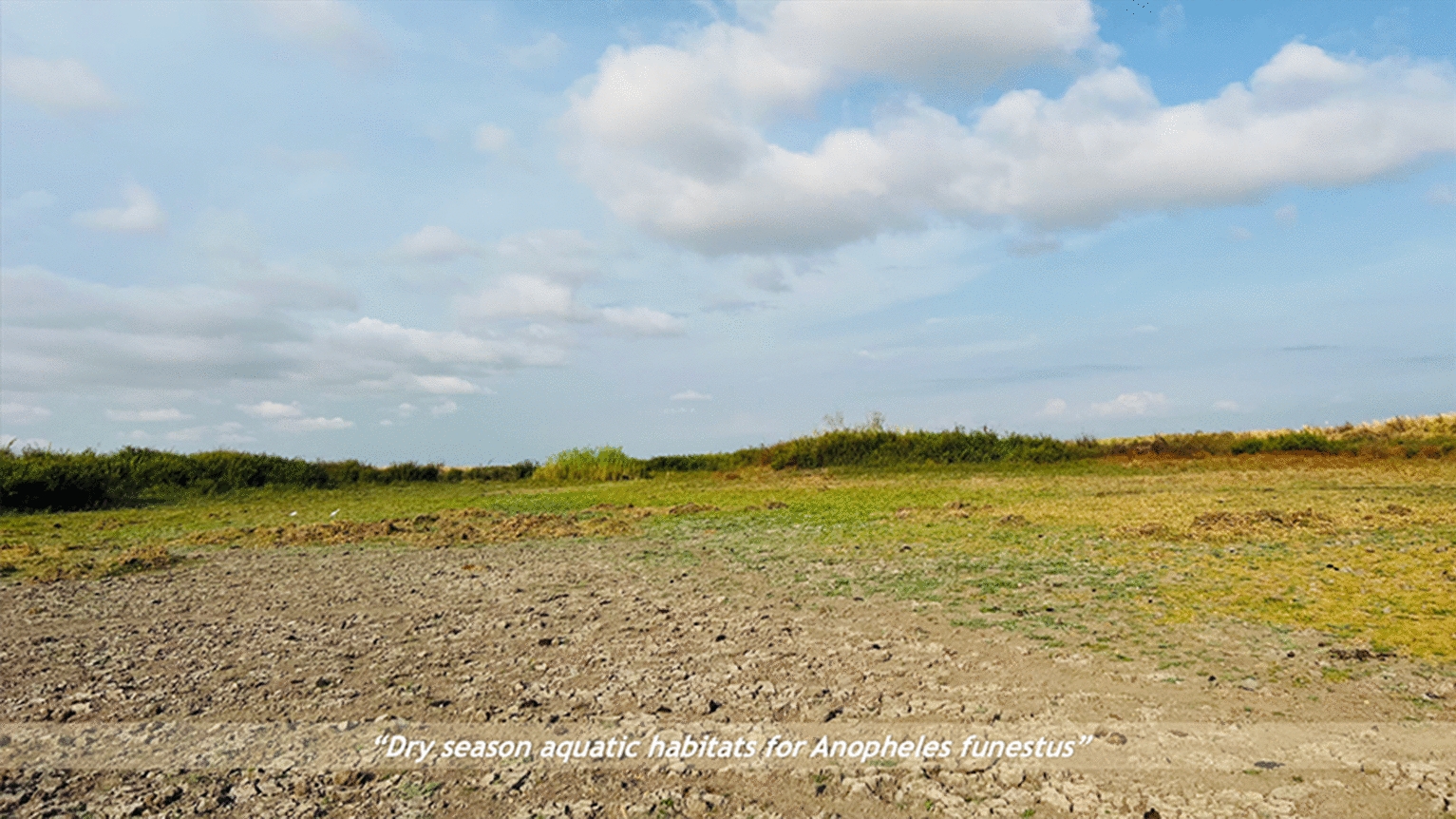

## Background

Malaria control strategies have primarily focused on insecticide-treated nets (ITNs) and indoor residual spraying (IRS) to combat mosquito vector populations. However, these interventions are currently facing major challenges, including widespread insecticide resistance and behavioural adaptations of vector species [1, 2]. In response, there is need for complementary interventions, including larval source management (LSM), which is increasingly being considered by endemic countries, particularly in urban and peri-urban settings [1, 3]. In rural areas, LSM often faces various logistical challenges, particularly due to the complexity of aquatic habitats. Existing guidelines suggest that the aquatic habitats must be 'few, fixed and findable' [4], yet in most endemic regions this is rarely the case. The sheer numbers of breeding sites as well as their dynamic nature and inaccessibility illustrate the gap between LSM guidelines and their practical implementation in diverse settings.

Successful implementation of LSM programs requires a thorough understanding of the larval ecology of the target species, and especially the ability to locate their main aquatic habitats [5]. Whether malaria transmission is seasonal or perennial, identifying the main habitats that sustain vector populations throughout the dry seasons would be particularly important since such habitats could be targeted to maximize control when the vector populations are lowest [6].

*Anopheles funestus* sensu stricto (*Anopheles funestus* s.s.) is widely recognized as a major malaria vector in Eastern and Southern Africa [7, 8]. In south-eastern Tanzania [9, 10], as well as in some districts of northern Tanzania [11], this species is now responsible for > 85% of malaria transmission. This dominance is due to several attributes of this mosquito species, including its preference for both feeding on humans indoors and resting indoors [12, 13], its strong resistance to common pyrethroid insecticides [14] and its high daily survival rates [12]. Indeed, field evidence suggests that *An. funestus* can dominate malaria transmission even in areas where its densities are lower than those of other malaria vector species [9]. Unfortunately, in many settings, its basic biology and ecology are less well characterized compared to those of other vector species [8].

While studies focusing on *An. funestus* larval ecology are scarce, some of the studies carried out so far show that whereas its aquatic habitats occasionally overlap with those of other mosquito species, *An. funestus* possesses certain unique attributes that underlie its preferences [15, 16]. Early studies in the 1930s provided valuable insights, indicating that *An. funestus* was more likely to be found in permanent water bodies, such as river streams, ditches and ponds [17, 18], unlike *An. gambiae* complex mosquitoes, which generally prefer smaller and less permanent habitats [19]. A more recent study in south-eastern Tanzania found that *An. funestus* primarily oviposits in habitats along river tributaries, and in large ponds [17]. Distinctive features of these habitats, compared to those used by other malaria vectors, included clear waters, emergent vegetation, shading, water depths exceeding 0.5 m and permanent or semi-permanent availability [17]. Given the significance of *An. funestus* in the region, there is a need to extend these efforts by conducting detailed analyses of the importance of land cover characteristics.

The current study was therefore designed to explore how habitat characteristics, land cover types and human population densities affect *An. funestus* distribution, and then to use the findings to create habitat suitability maps for the vector species in south-eastern Tanzania.

## Methods

### Study site

**Fig. 1:**
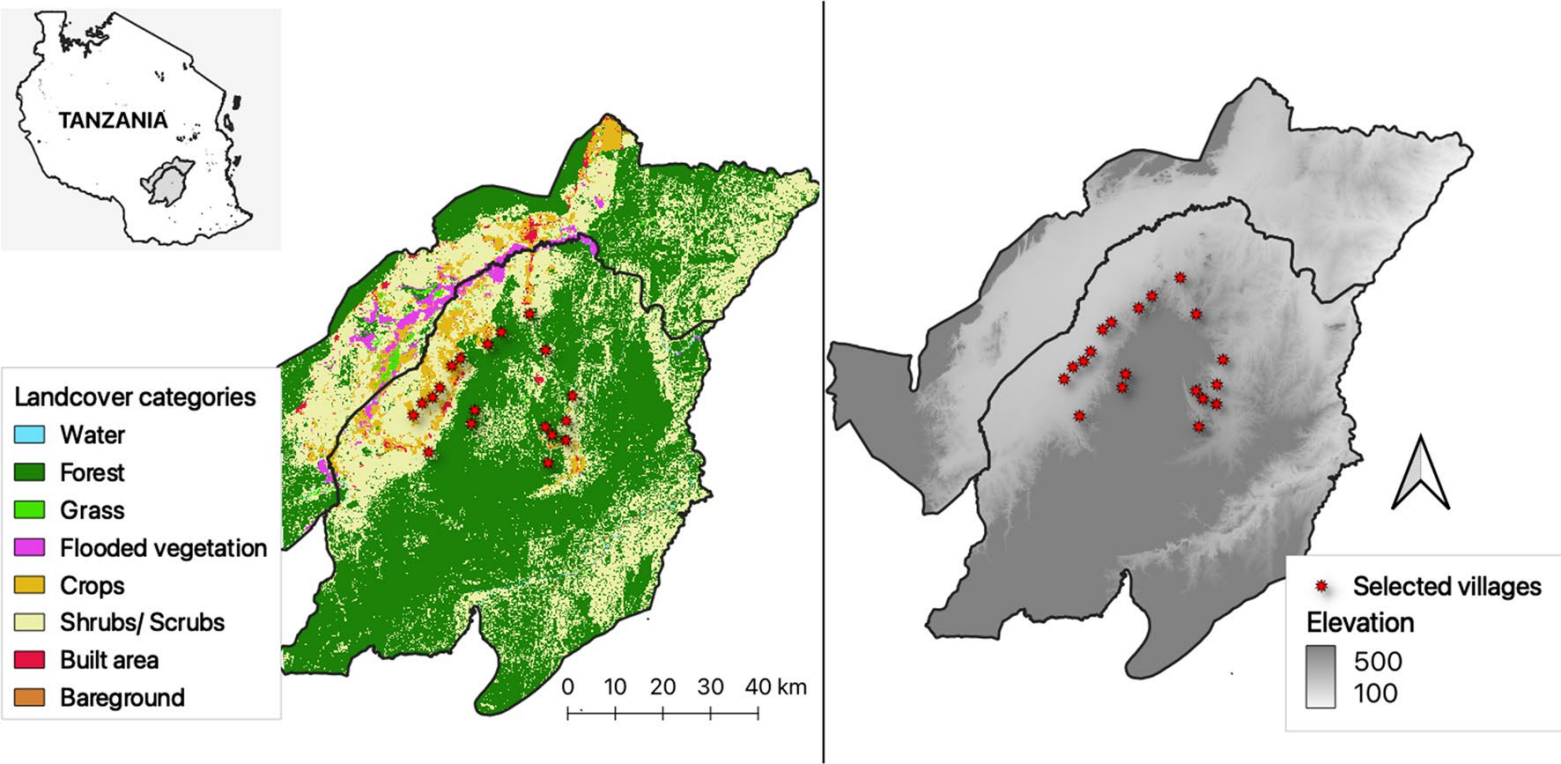
Study villages (filled red circles) for the dry season surveys of *Anopheles funestus* aquatic habitats in south-eastern Tanzania

The field survey was conducted in 18 villages located in south-eastern Tanzania, including 11 villages in Ulanga district (Chikuti, Chirombora, Ebuyu, Gombe, Ikungua, Iragua, Kichangani, Kidugalo, Lukande, Mwaya and Mzelezi) and seven villages in Malinyi district (Itete, Mtimbira, Sofi Mission, Sofi Majiji, Kalengakelo, Kiswago and Ipera Asilia) in south-eastern Tanzania (Fig. 1). The area has an altitude of 250–650 m a.s.l., yearly mean temperature ranges of 20–33 °C and annual rainfall range of 1200–1800 mm [20]. Generally, the dry season occurs between June and November, short rains occur in November and December, and long rains occur from February to May [20]. The area has diverse land use features, including small towns, villages, savannahs, crops, irrigation, grazing lands, forests and shrublands. There is a large flood plain with numerous rice farms, bordered by Udzungwa mountains to the north and Mahenge hills to the south (Fig. 1). *Anopheles arabiensis* and *An. funestus* are the main malaria vectors, with the latter species mediating most of the transmission [9, 21]. The main economic activities are livestock-keeping, fishing and crop farming [22, 23].

### Sampling and characterization of aquatic habitats

The habitat survey was conducted during the dry months of September to December 2021. Community members aged ≥ 18 years were recruited and trained to identify potential aquatic habitats, including natural and human-made water bodies, and to record their physico-chemical attributes, regardless of whether mosquito larvae were present or not. To ensure a comprehensive coverage of each village, a team of five people walking at a distance of 2 m from each other systematically surveyed the area along pre-set transects, within demarcations set by the village authorities.

For each water body observed during the transect walks, the team recorded: (i) time and date of visit; (ii) GPS coordinates; (iii) habitat type (classified into river streams, stagnant ground pools, marshes, wells, dug pits, brick or concrete pits, ditches, rice fields, hoofprints); (iv) habitat size (surface area); (v) water clarity; (vi) water source (rainfall accumulation or ground water); (vii) water movement (stagnant, slow or fast moving); (viii) water permanence (permanent, semi-permanent, or temporary); (ix) water depth; (x) presence and type of algal growth (brown, blue, filamentous); (xi) presence of shading; (xii) types and quantity of vegetation; and (xiii) environmental characteristics surrounding the habitats within 200 m (such as cultivation, bush areas, cattle grazing and distance to nearest human habitations). Additionally, physico-chemical metrics, including pH, total dissolved solids (TDS) and electroconductivity (EC), were measured using a water-quality meter.

### Larval surveys

All identified water bodies were examined for the presence of mosquito larvae using either the standard 350-ml dipper (for small habitats with shallow depths) or a large 10-l bucket (for larger and deeper habitats), as previously described [17]. The number of dips performed in each aquatic habitat was determined based on its size, following a predefined protocol. For habitats < 5 m^2^, a single dip was made; in habitats measuring 6 to 10 m^2^, two dips were made; and for those habitats ranging in size from 11 to 15 m^2^, three dips were made. This incremental approach continued for larger habitats, with a limit of 20 dips for any habitat > 120 m^2^. Collected larvae were identified to genus and species group level, whenever possible, using standard taxonomic keys [24, 25]. Within the *Anopheles* genus, late instars (III and IV) of the *An. funestus* group and the *An. gambiae* complex could be easily distinguished based on their morphology [25, 26]. Consequently, in this article, the term “aquatic habitats” refers to any surveyed water body, while “positive habitats” denotes those where *An. funestus* was confirmed via dipping.

### Environmental covariates

A digital elevation model (DEM) with 10-m resolution [27] was used to extract data on elevation, slope, terrain and aspect for each aquatic habitat location. Land cover data were derived from the European Space Agency (ESA) Sentinel-2 satellite imagery acquired in June 2022. These data consisted of eight land cover classes and had a spatial resolution of 10 m, with and an overall accuracy of 75% [28]. The ESA imagery allowed analyses of both land cover and land use characteristics, such as urban areas and forestation, and helped identify small-scale landscape features and patterns crucial for understanding the local level relationships with malaria risk [29]. For each aquatic habitat, the proportion of each land cover type (water, trees, grasslands, flooded vegetation, shrubs, built-up areas, bare ground and crops) was extracted within a 300-m buffer. In addition, the distance from each aquatic habitat to the nearest feature of each land-cover class was measured. Consideration of both the buffer zone and distance to habitats allowed for a more nuanced analysis of how both the immediate landscape composition and the proximity to specific land cover types correlate with the presence of *An. funestus* larvae in the aquatic habitats.

Finally, human population densities data within the 300-m buffer were obtained from the Global Human Settlement Layer (GHSL) project, a spatial raster dataset composed of 100 × 100-m cells, with each representing the number of people in that area [30].

### Statistical analysis

**Fig. 2:**
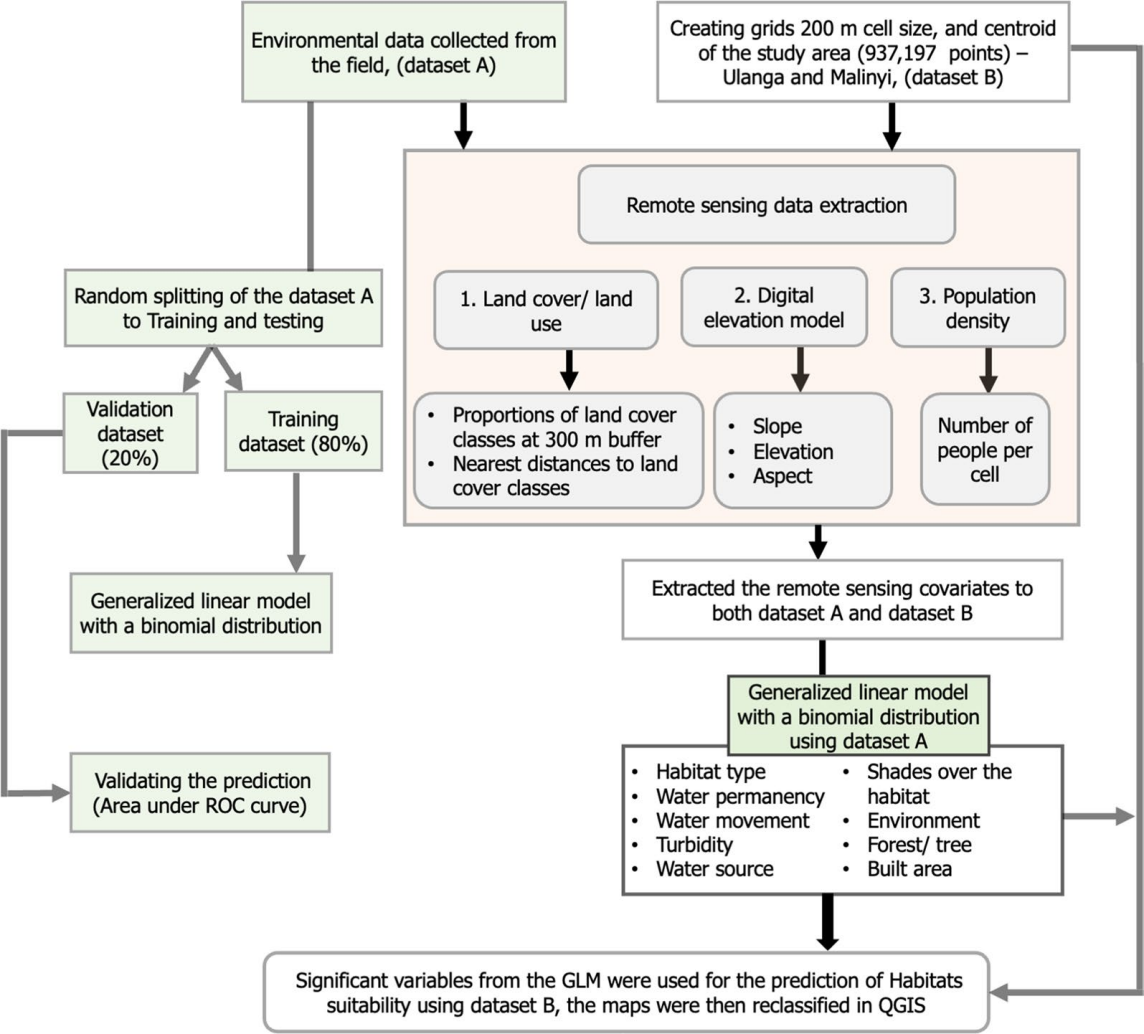
Flowchart showing the analysis procedures, involving two datasets. On the left side, the boxes highlighted in green represent the field-collected data (dataset A), modelled using logistic regression to determine the significant variables and validated using the two-fold cross-validation technique. On the right side is a 200-m grid covering the entire study area (dataset B), which was used to perform the prediction of the habitat’s suitability based on the retained significant variables

An initial descriptive analysis was conducted to assess the occurrence and distribution patterns of aquatic habitats occupied by *An. funestus*, as well as variations by type, specific location (village) and land cover categories (Fig. 2). A multivariate generalized linear model (GLM) with a binomial distribution was then used to examine the relationship between the presence of *An. funestus* larvae (absent = 0; present = 1) and a range of environmental and landscape variables (Table 1). Starting with a full model, including all the candidate variables, an automated backward stepwise selection was used to identify significant variables for inclusion in the final model, based on likelihood-ratio-tests (Table 1). To quantify the strength of association between the presence of *An. funestus* larvae and these variables, odds ratios (ORs) were calculated as part of the GLM. The ORs provide a measure of the likelihood of larvae presence associated with each variable.

**Table 1:** Candidate covariates evaluated for predicting the presence of aquatic habitats of *An. funestus* mosquitoes

Variable	Source of data and description of variables
Village	Physical parameter recorded for each aquatic habitat in the field
Habitat type^a^	
Habitat size	
Water depth	
Water source^a^	
Watercolor^a^	
Water movement^a^	
Permanence of water^a^	
Presence of vegetation	
Vegetation types	
Algae status	
Types of algae	
Presence of shade^a^	
Surroundings environment^a^	
Distance to nearby house	
*Proportion of:*	Proportion of land cover classes calculated in a 300-m buffer around each aquatic habitat from the surveyed points Data from European Space Agency (ESA) Sentinel-2 satellite imagery [28]
- tree/forest areas^a^	
- shrublands	
- grassland	
- crops	
- built-up areas^a^	
- bare land	
- flooded vegetation	
*Distance (m) from the nearest:*	Distances (m) between each aquatic habitat and the nearest patch of each land cover class Data from European Space Agency (ESA) Sentinel-2 satellite imagery [28]
- tree/forest	
- shrubland	
- grassland	
- crops	
- built-up area	
- bare land	
- flooded vegetation	
Elevation (m)	Derived from a 10-m resolution digital elevation model (https://earthexplorer.usgs.gov) [27]
Slope (°)	
Aspect	
Population density	Number of people living in the 300-m buffer Data obtained from the Global Human Settlement Layer project [30]

^a^Significant variables that were retained in the final model (details provided in Results section)

A two-fold cross-validation process was used to validate performance of the model. The dataset was divided into a training subset (80%) and a test subset (20%). The model was trained on the training dataset and validated on the test set using Tjur's* R*^*2*^ calculations and area under the curve (AUC) receiver operating characteristics (ROC), with upper limits of 1.0 for a perfectly fitting model (Fig. 2).

The final model was used to generate spatial predictions of the likelihood of encountering *An. funestus* larvae in aquatic habitats found in different locations. To generate these maps, we created a 200-m resolution grid of regular points covering the study area of Malinyi and Ulanga districts (total area = 22,777 km^2^). Covariates retained in the final logistic model, such as proportions of land cover types, were extracted and applied to each grid to predict habitat suitability across the unsampled areas. To model how variations in specific habitat characteristics might influence the suitability for *An. funestus*, multiple scenarios were tested, with varied attribute values. For example, scenarios were created where water turbidity or habitat permanence were varied, reflecting different potential conditions.

All statistical analyses, including variable extraction, model fitting and predictions, were performed using the R statistical program version 4.2.1, with the packages rms, MASS, lme4 and glmm [31]. Preliminary data handling and visualization were performed using the software QGIS (Quantum Geographic Information System [32]).

### Interactive maps for predictions and web application

To facilitate the exploration of different suitability scenarios, we also developed an interactive map using the Leaflet and Shiny packages in R [33] This web-based tool provides a dynamic platform for viewing and adjusting predictions from our model through a user-friendly graphical user interface (GUI). The GUI is designed to be intuitive, allowing different stakeholders to interactively modify model inputs and observe the effects on the geographical suitability for *An. funestus* habitats. Users can select or alter the values of various parameters, then update the predictive maps to instantly visualize how these changes affect the predicted suitability.

## Results

### Descriptive analyses of *An. funestus* positivity in different habitat type

**Fig. 3:**
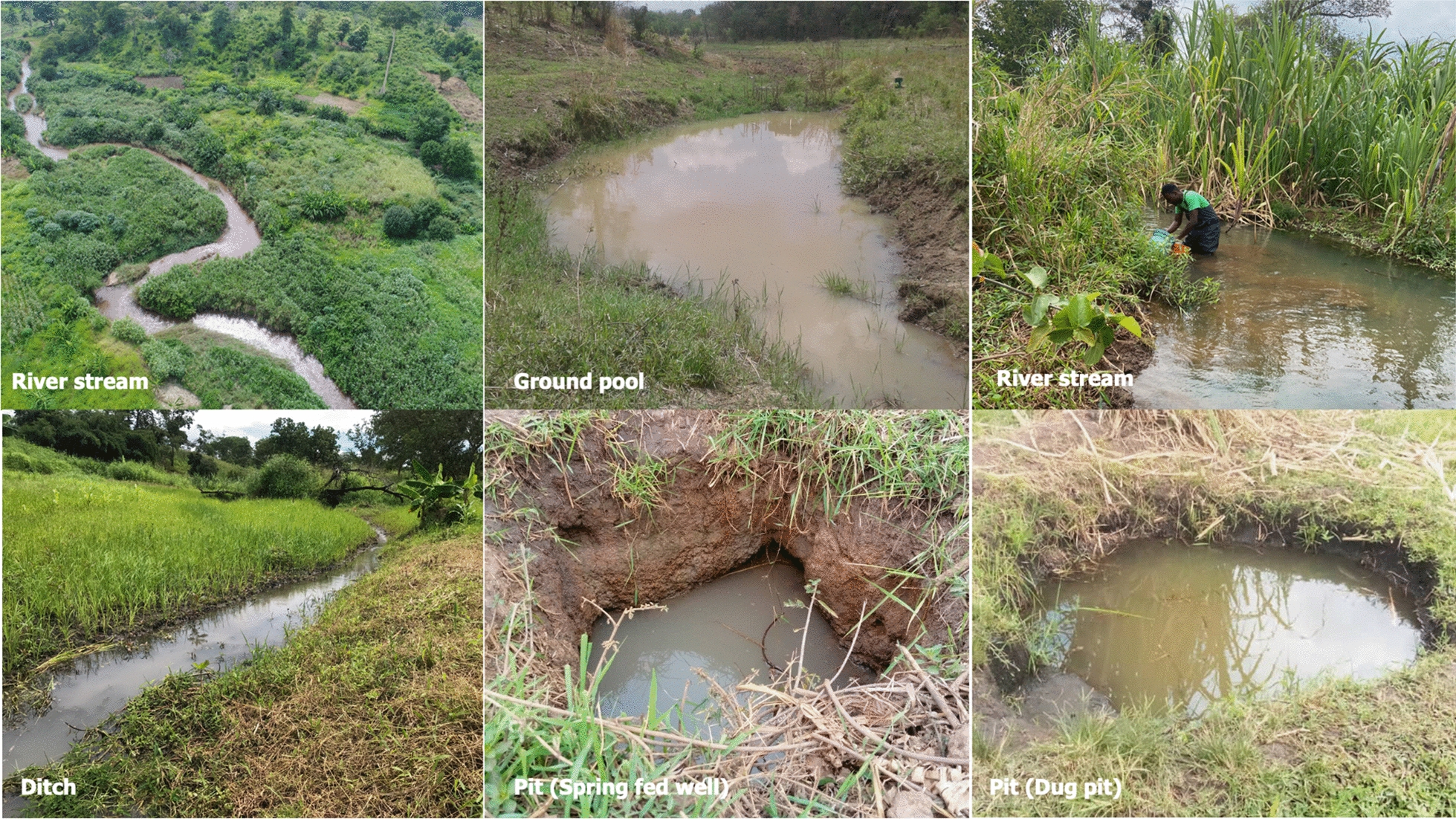
Example of surveyed aquatic habitats that were found to harbour *Anopheles funestus* larvae

The comprehensive assessment of potential habitats included river streams, ground pools, wells, dug pits, brick and concrete pits, rice fields and ditches (Fig. 3). Among the 1466 potential aquatic habitats inspected, 440 (30%) were positive for *An. funestus* larvae. River streams were the commonest water bodies observed, accounting for 695 of the 1466 habitats inspected, and approximately three-quarters of these had *An. funestus* larvae (Table 2). Ground pools had the next highest positivity for *An. funestus* larvae (15% of 212 habitats), followed by wells and dug pits (4.8%), ditches (4.5%), rice fields and concrete pits (< 1.2%; Table 2). Notably, no *An. funestus* larvae were detected in the 10 hoofprint habitats surveyed. Puddle or vehicle tracks were not present at all.

**Table 2:** Percentage of habitats of different types that had *Anopheles funestus* larvae

Habitat type	Total number of each habitat type	Percentage of habitat type with *An. funestus* larvae^a^
River streams^b^	695	74.0
Ground pools^c^	212	15.0
Spring-fed wells and dug pits^d^	409	4.8
Brick and concrete pits^d^	49	0.6
Agricultural fields/rice fields	27	1.1
Ditch	68	4.5
Hoofprint	10	0.0
Puddle and tyre track	0	–
Total	**1466**	**100**

^a^Values are averaged across villages

^b^During the survey, river streams were divided into 50-m-long segments, and each segment was individually characterized. This habitat class also included remnant water pools on the riverbeds and, therefore, multiple segments could be part of the same river stream

^c^Ground pools included large or small marshes and ponds with stagnating water, sometimes with vegetation present

^d^Habitats defined as pits included spring-fed wells and dug pits, as well as brick and concrete pits, and all were created by communities

### Descriptive analysis of *An. funestus* habitat types in different villages

The percentage of *An. funestus* aquatic habitats varied between villages, likely in association with local differences in habitat types. For example, in Chikuti, *An. funestus* larvae were found exclusively in river streams, accounting for 100% of positive habitats. Similarly, in Lukande and Mtimbira villages, river streams accounted for 97% and 96% of all positive habitats, respectively. However, there were also villages where river streams were present but completely lacked *An. funestus* larvae (e.g. Kichangani and Ipera Asilia). Ground pools, the habitat type with the second highest *An. funestus* positivity, accounted for 100%, 85% and 60% of all positive habitats in Ipera Asilia, Mwaya and Sofi Mission villages, respectively. On the other hand, rice fields, ditches and hoofprints appeared to be unfavorable for *An. funestus* larvae, with either minimal presence or complete absence of the vector species (Table 3).

**Table 3:** The percentage of habitats of different types positive for *Anopheles funestus* in different villages in Ulanga and Malinyi districts during the dry season of 2021

Village	Total number of habitats counted	*An. funestus*-positive habitats (*n*)	Total number of habitats observed (and percentage positive for *An. funestus*)					
			River streams	Ground pools	Pits^a^	Rice fields	Ditches	Hoofprints
Chikuti	47	9	23 (100)	2 (0)	22 (0)	0 (0)	0 (0)	0 (0)
Chirombora	85	34	37 (85)	12 (15)	36 (0)	0 (0)	0 (0)	0 (0)
Ebuyu	88	44	44 (84)	7 (11)	35 (5)	0 (0)	0 (0)	2 (0)
Gombe	86	42	61 (83)	3 (7)	14 (2)	1 (0)	5 (7)	2 (0)
Ikungua	213	66	85 (35)	31 (27)	24 (7)	21 (7)	52 (23)	0 (0)
Ipera Asilia	11	6	5 (0)	6 (100)	0 (0)	0 (0)	0 (0)	0 (0)
Iragua	64	9	28 (67)	14 (0)	16 (22)	3 (0)	3 (11)	0 (0)
Itete	38	26	26 (80)	3 (8)	9 (12)	0 (0)	0 (0)	0 (0)
Kalengakelo	119	30	66 (87)	4 (3)	49 (10)	0 (0)	0 (0)	0 (0)
Kichangani	57	0	33 (0)	9 (0)	15 (0)	0 (0)	0 (0)	0 (0)
Kidugalo	85	17	37 (82)	2 (6)	45 (12)	0 (0)	0 (0)	1 (0)
Kiswago	19	12	8 (58)	3 (25)	8 (17)	0 (0)	0 (0)	0 (0)
Lukande	111	35	56 (97)	13 (0)	40 (0)	1 (0)	1 (3)	0 (0)
Mtimbira	108	27	48 (96)	1 (0)	53 (4)	0 (0)	1 (0)	5 (0)
Mwaya	102	13	36 (15)	50 (85)	14 (0)	1 (0)	1 (0)	0 (0)
Mzelezi	68	36	61 (94)	0 (0)	5 (6)	0 (0)	2 (0)	0 (0)
Sofi Majiji	67	14	16 (86)	9 (14)	42 (0)	0 (0)	0 (0)	0 (0)
Sofi Mission	98	25	25 (40)	43 (60)	27 (0)	0 (0)	3 (0)	0 (0)

^a^Habitats defined as pits included spring-fed wells and dug pits, as well as brick and concrete pits, and all were created by communities

### Descriptive analyses of *An. funestus*-positive habitats in areas with different land cover types

Tree covered areas were the most abundant land cover type within the surveyed area, and were also the land cover type with the highest number of aquatic habitats (Table 4). Other land covers, including grasslands, shrublands and agricultural fields, also had significant numbers of aquatic habitats. In contrast, built-up areas showed a markedly lower presence of aquatic habitats suitable for *An. funestus* larvae.

**Table 4:** Area in square kilometres and the percentage of each land cover type, number of aquatic habitats found in each land cover category and number and percentage of habitats occupied by *An. funestus* larvae

Land cover category	Area of land cover, in km^2^ (%)	Number of aquatic habitats found in each land cover category	Number of *An.* *funestus*-positive habitats in each land cover category (%)
Trees and forests	14,277 (63.0)	486	194 (40)
Shrubland	6765 (30.3)	399	108 (27)
Grassland	59 (0.3)	55	13 (24)
Crops/agricultural fields	841 (3.8)	380	75 (20)
Built areas	84 (0.4)	129	16 (12)
Flooded vegetation	163 (0.7)	11	1 (9)
Bare land/open space	41 (0.2)	0	-
Water bodies	116 (0.52)	0	-

### Environmental predictors of *An. funestus* presence in aquatic habitats

The *R*^2^ of the final model was 0.28, indicating modest explanatory power but with high accuracy (AUC of the final model = 0.83) [34]. Among the 33 environmental and landscape variables investigated, nine were retained based on statistical significance in the final model: habitat type, water movement, water clarity, water source, permanence of the habitat, shading over habitats, presence of algae, tree cover and built-up area in a 300-m buffer zone.

Regarding habitat types, pits and those classified as 'other' showed lower odds of hosting *An. funestus* larvae compared to natural river streams. Although ground pools showed a higher occurrence of larvae than river streams, this difference was not statistically significant (Table 5). *Anopheles funestus* larvae were less frequently found in stagnant (OR = 0.42, *P* < 0.001) and unclear water sources (OR = 0.67, *P* = 0.02) compared to clear, flowing waters. Notably, *An. funestus* preferred permanent as opposed to temporary habitats such as those formed from rainwater accumulation. Shaded habitats and algal absence were also positively associated with the occurrence of *An. funestus* larvae.

**Table 5:** Results of multivariate generalized linear model of habitat suitability for *An. funestus* habitats

Characteristics	Odds ratios (95% CI)	*P*-value^a^
*1. Habitat type*		
River streams	1	
Ground pools	1.32 (0.85, 2.04)	0.20
Pits	0.25 (0.14, 0.43)	< 0.001***
Others	0.29 (0.16, 0.53)	< 0.001***
*2. Water movement*		
Moving	1	
Stagnant	0.42 (0.29, 0.59)	< 0.001***
*3. Water clarity*		
Clear	1	
Unclear	0.67 (0.48, 0.93)	0.02*
*4. Water source*		
Non rainwater	1	
Rainwater	3.65 (2.57, 5.16)	< 0.001***
*5. Water permanency*		
Permanent	1	
Semi-permanent	0.25 (0.14, 0.42)	< 0.001***
*6. Shading over habitat*		
None	1	
Shaded	1.45 (1.08, 1.96)	0.015*
*7. Algae status*		
None	1	
Present	0.64 (1.54, 4.18)	< 0.001***
*8. Environment*		
Cattle grazing	1	
Cultivated field	1.83 (0.88, 3.79)	0.07
Scrub	1.37 (0.65, 2.89)	0.40
Mixed	2.55 (1.25, 5.18)	0.008**
Land cover significant parameters		
*9. Proportion of trees at 300-m buffer*	2.83 (1.73, 4.62)	< 0.001***
*10. Proportion of built-up area at 300-m buffer*	0.34 (0.12, 0.98)	0.025*

Data in table are the odds ratios with 95% lower and upper confidence intervals (CI) and the* P*-values of the variables retained in the best model

^a^Significance at *P < 0.05, **P < 0.01 and ***P < 0.001

With respect to land cover types, *An. funestus* larvae were more likely to be found in aquatic habitats situated in areas with extensive tree cover and forest canopies (OR = 2.83, *P* < 0.001). In contrast, the presence of *An. funestus* larvae was negatively associated with habitats within or near built-up areas (OR = 0.34, *P* = 0.025). Finally, no significant associations were observed between the presence or absence of *An. funestus*-positive habitats and either human population densities or the different landscape factors derived from the digital elevation model.

### Predicting suitability for* An. funestus* larvae presence

**Fig. 4:**
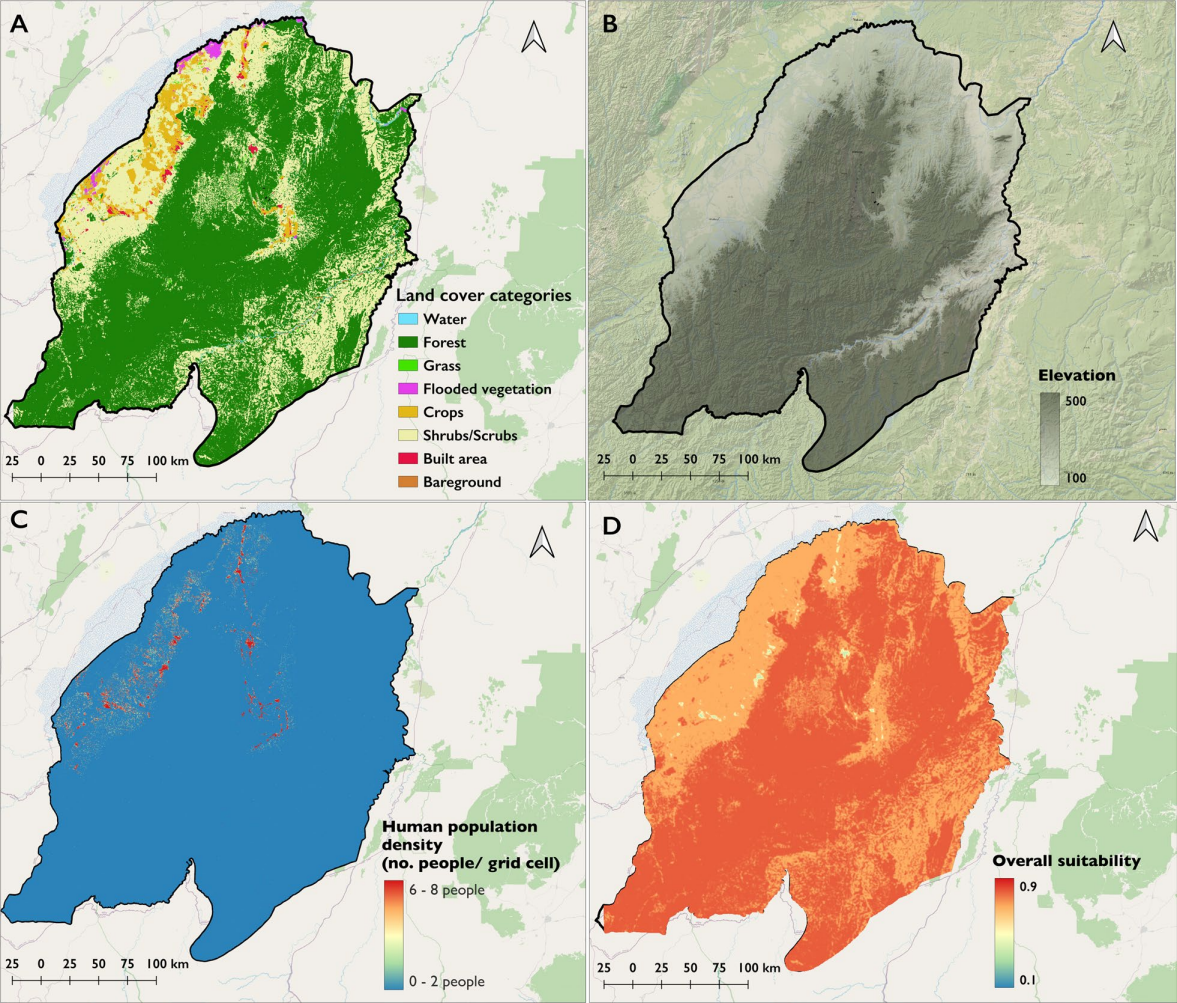
Map showing the overall suitability for *An. funestus* larval habitats and visualization of the other remote sensing predictors. **a** Land cover, presents a classification of the area land cover, **b** terrain elevation of the area, with grey shading indicating the gradient from lower to higher elevations, **c** density of human population per grid cell, with a colour gradient from blue to red, where blue represents lower density areas and red represents higher density areas, **d** overall suitability—a synthesis of the significant remote sensing and habitat characteristics data into an overall suitability map for *An. funestus* larval habitats

The final model was used to predict the expected suitability for *An. funestus*-positive habitats throughout the entire study area, including villages from where no field surveys had been conducted (Fig. 4).

**Fig. 5:**
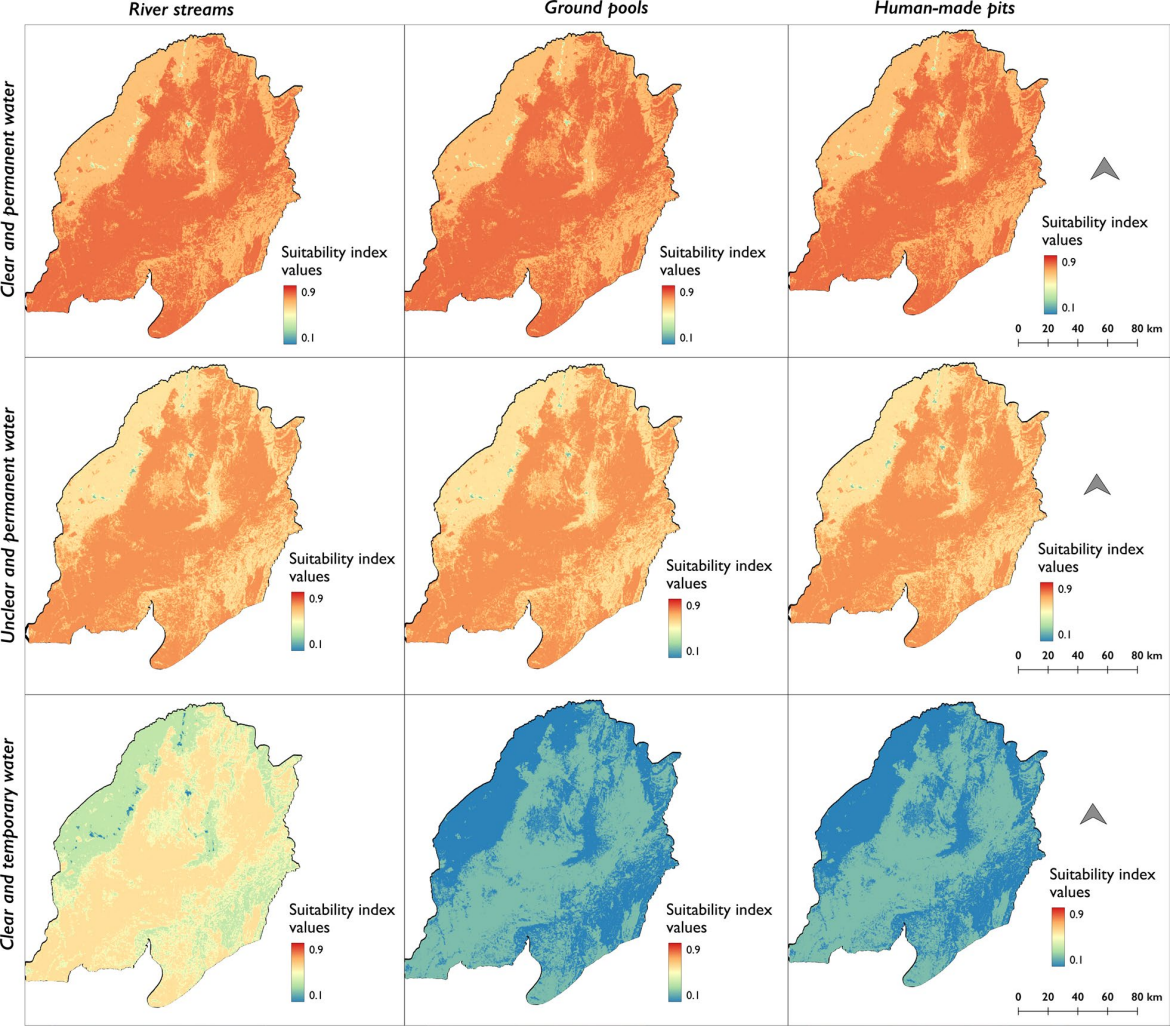
Examples of suitability maps for *An. funestus* larval habitats under diverse environmental scenarios. This figure serves as an illustrative tool for understanding the varying suitability of larval habitats for *An. funestus* across different environmental scenarios. These examples show how specific conditions, such as water clarity, permanence and habitat type (river streams, ground pools, and pits) influence habitat suitability, while other environmental conditions remain constant (moving water, rainwater as water source, shaded habitats, absence of algae, areas with cattle grazing, cultivated fields and scrubs)

Due to the variability of environmental factors at different scales, we used an interactive system in which different scenarios of how specific environmental conditions might influence the distribution and suitability of habitats for *An. funestus* can be visualized and evaluated. Figure 5 shows examples of scenarios reflecting both the likelihood of an *An. funestus* habitat being present and the importance of specific conditions of the individual habitats. This multifaceted approach allowed us to explore the spectrum of environmental conditions and their impacts on the presence of *An. funestus* larvae, under the assumption that aquatic habitats are present at these locations. Notably, the central region of the study zone consistently shows the highest suitability for *An. funestus* habitats, attributable to factors such as dense tree cover, persistent water bodies, clear and flowing water and shading conditions, all of which are ideal for habitation by this vector species. In contrast, areas closer to built-up regions with temporary, unclear and stagnant waters shows lower suitability.

Ground pools and river streams, for example, generally exhibit higher suitability compared to pits. Further visualization is facilitated through an interactive web application, which can be accessed online for a comprehensive exploration of these environmental impacts on habitat suitability at http://boydorr.gla.ac.uk/lucanelli/kahamba_funestus/.

## Discussion

The present investigation on the aquatic ecology of *An. funestus*, particularly during the dry season, addresses a critical gap in LSM strategies. In this study, we investigated the associations of habitat characteristics and different land covers on the presence and distribution of this vector species in two south-eastern Tanzania districts. Our findings reveal that *An. funestus* larvae predominantly inhabit river streams and ground pools, with a marked preference for areas characterized by extensive tree cover, grasslands and shrublands. Conversely, larvae are less frequently found in built-up and semi-urban areas. Additionally, the study findings highlight the species’ adaptability, showing that they thrive in both vegetated and non-vegetated habitats, and their preference for the more permanent water bodies over temporary ones.

*Anopheles funestus* larvae were found in various habitat types, including river streams, ground pools and pits such as spring-fed wells and dug pits. In the dry season, we observed that some river streams undergo evaporation, resulting in the formation of isolated pools in the river stream bed. In some areas, local communities intentionally created artificial pools by blocking the natural flowing of river streams for the purpose of agricultural irrigation, and these became important habitats for *An. funestus*. Other pits, designed for various purposes, such as construction and domestic use, also served as larval habitats, as previously observed [35]. Large ground pools characterized by stagnant water and emergent vegetation were also found. Observations that river streams are an important feature of *An. funestus* ecology has previously been reported both in Eastern and Western Africa [36, 37]. Similarly, habitat stability and vegetation cover have also been reported as important factors affecting the vector species. For example, in one study in western Kenya near Lake Victoria, *An. funestus* larvae were found only during periods of high water levels [18]; and in southern Mozambique, the species was most abundant in vegetated swamps where water accumulated throughout the year [18].

This study also identified a higher likelihood of finding *An. funestus* larvae in clear aquatic habitats compared to unclear habitats, which is consistent with previous research in the same settings [17], and in western Kenya highlands, where high prevalence of *An. funestus* larvae were seen in clean water bodies [16, 38, 39]. Debrah et al. and Nambunga et al., both of whom carried out studies in rural communities, emphasized the importance of proximity to human dwellings as a significant factor for *An. funestus* habitation [16, 17]. In our study, where this factor was observed from the perspective of land cover, we found that the likelihood of *An. funestus* habitation was lower in built-up areas with concentrated human infrastructure, suggesting that anthropogenic effects on the environments negatively impact the ecology of *An. funestus*. Moreover, our expansive methodology meant that even the most remote locations were inspected, allowing us to identify aquatic habitats far from residential areas. These remote habitats should not be ignored as they could serve as key refugia that support the persistence of mosquito populations [40].

Our study, conducted in the dry season, found that most aquatic habitats for *An. funestus* were permanent, corroborating earlier research by Mwangangi et al. [36], Nambunga et al. [17] and others [16, 41]. However, a key limitation to these earlier studies is that the habitats were not tracked across different seasons. To fully understand habitat permanence and its impacts on *An. funestus*, year-round monitoring across both wet and dry seasons is necessary. It therefore remains uncertain if these mosquitoes prefer the permanent habitats all year-round or if these are simply the only option available in the dry season. Furthermore, contrary to reports in previous studies of *An. funestus* having a preference for dense vegetation in habitats [15, 16], our observations indicate that this species is capable of laying eggs in both vegetated and non-vegetated habitats, with a particular affinity for habitats rich in algae. The occurrence of *An. funestus* larvae in habitats with algae suggests a potential symbiotic relationship between the larvae and algal blooms, possibly driven by the nutrients provided by the algae [42, 43].

The results of the present study revealed localized differences in aquatic habitat distribution across several villages, underscoring *An. funestus*'s selective habitat use even within small geographical areas. *Anophels funestus*-positive river streams, for example, were common in Chikuti, Lukande and Mtimbira, but not in Kichangani and Ipera Asilia, indicating that habitat suitability is affected by the unique characteristics of each river and its ecosystem. Similar patterns were observed in other studies, highlighting the complexity of *An. funestus* ecology [16, 36] and emphasizing the understanding that not all habitats, even within the same category, can support *An. funestus* larvae [44]. Habitats like rice fields, ditches and hoofprints showed little or no presence of *An. funestus* larvae, aligning with earlier research suggesting their selective breeding site preferences, often avoiding human-modified habitats [16]. This underscores the need for localized research and tailored interventions [45], as effective strategies in one area might not be suitable in another due to variations in habitat preferences [6].

This study also shows that *An. funestus* predominantly inhabits pristine environments, such as forests, grasslands and shrublands, and is found less common in human-modified areas, such as urban or semi-urban settings. A significant association was found between *An. funestus* larvae and natural land cover types, with forested regions providing shaded, humid microclimates being conducive to larval survival [46, 47]. Factors such as shade from tree canopies and favourable microclimates enhance larval persistence as well. Moreover, river streams, which were the dominant habitat type in these tree-covered areas, had the highest likelihood of larvae presence. In contrast, built-up areas and flooded areas limit the number of suitable breeding grounds, leading to reduced suitability for *An. funestus* larvae [48, 49]. This negative association with built-up areas may be attributed to the environmental management and infrastructural development processes in these settings, which may reduce the availability of conducive breeding grounds for *An. funestus*.

Although the larval ecology of *An. funestus* has not been extensively studied [8], and its relationship with land cover needs more exploration, existing research, including studies in Kenya, supports our findings of an association between forested areas and larvae presence [38, 50]. The present study, along with other earlier investigations, identifies various land cover types, such as forests, farmland and pastures, as potential habitats, underscoring the adaptability of *An. funestus *[39, 49]. These variations also underline the importance of understanding how different environmental and land cover factors contribute to the distribution of *An. funestus* larvae, which is vital for implementing effective LSM strategies and malaria control programs [6, 51]. Targeting specific habitats that are hotspots for *An. funestus* larvae allows for a more efficient allocation of resources and implementation of interventions such as larviciding or habitat modification [4].

The results of our study suggest that the species uses clear, permanent and shaded water environments. However, this study recognizes that these habitats can vary in their characteristics over time. For example, habitats that are typically clear might become unclear and polluted at times, and even flowing waters may become stagnant during certain seasons. In contrast, *An. gambiae* sensu lato is known for its ecological adaptability. This species can breed in a wider range of habitats, including both temporary and permanent water bodies [38, 52], and it can use breeding habitats such as puddles and agricultural fields which* An. funestus* often tends to avoid or use less frequently [53].

This study has a number of limitations that should be noted. First, while our model accounted for a significant portion of the variability in *An. funestus* habitation, it may have omitted other influential factors. To enhance the understanding of the ecology of *An. funestus*, future research could include variables such as the NDVI (Normalized Difference Vegetation Index), NDWI (Normalized Difference Water Index) and rainfall, which would provide some understanding on vegetation health and water body dynamics [54]. Moreover, including hydrological and geomorphology parameters could further provide more detailed insights into the physical environment and *An. funestus* distribution [55–57]. Second, the accuracy of detecting mosquito larvae is influenced by sampling methods, including the number of samples, technical expertise and spatial coverage. Our study may have been limited by the pre-specified nature of these parameters. Finally, integrating field environmental data with remote sensing land cover data presents multiple challenges. For example, the resolution of land cover data used here might have been insufficient to capture fine details such as isolated residences and small water bodies, thereby impacting habitat suitability mapping. To address these limitations, future studies may consider adopting higher-resolution remote sensing data sources, such as unmanned aerial vehicle (UAV) imagery [58–60], to capture finer details of habitats within a smaller geographical area and detect potential water sources for *An. funestus* [60].

## Conclusions

This study comprehensively identified the main land use and environmental factors that influence larval habitat use by *An. funestus* during the dry season in southern Tanzania, where the species is the dominant vector of malaria transmission. We found that river streams and ground pools were the primary larval habitats during the dry season and that water bodies in forested areas, grasslands and shrublands are most likely to be positive for *An. funestus*. In contrast, larvae were least likely to be found in aquatic habitats which are in built-up and semi-urban areas. These insights are crucial for the strategic implementation of LSM strategies, particularly during the dry season when habitats are typically “few, fixed and findable.” The habitat suitability model developed here can be instrumental in pinpointing geographic areas where *An. funestus* larvae are most likely to be found, thereby facilitating targeted LSM deployment. Such targeted strategies, including larviciding and habitat modification, can be more effectively applied in high-risk zones identified through our model, thereby enhancing the efficacy of malaria control measures during the dry season. Building on these insights will further refine our understanding of mosquito dynamics, paving the way for enhanced strategies in malaria control and elimination.

## Data Availability

The dataset supporting findings is available upon a reasonable request directed to the corresponding author or the Ifakara health institute in Tanzania.

## Abbreviations

AUC: Area under the curve
DEM: Digital elevation model
EC: Electroconductivity
ESA: European Space Agency
GHSL: Global Human Settlement Layer
GUI: Graphical user interface
IRS: Indoor residual spraying
ITNs: Insecticide-treated nets
LSM: Larval source management
NDVI: Normalized Difference Vegetation Index
NDWI: Normalized Difference Water Index
QGIS: Quantum Geographic Information System
ROC: Receiver operating characteristics
TDS: Total dissolved solids
UAV: Unmanned aerial vehicle
