# Correction: Geospatial modelling of dry season habitats of the malaria vector, *Anopheles funestus*, in south-eastern Tanzania

**DOI:** 10.1186/s13071-024-06181-0

**Published:** 2024-02-13

**Authors:** Najat F. Kahamba, Fredros O. Okumu, Mohammed Jumanne, Khamisi Kifungo, Joel O. Odero, Francesco Baldini, Heather M. Ferguson, Luca Nelli

**Affiliations:** 1 Environmental Health and Ecological Sciences Department, Ifakara Health Institute, P. O. Box 53, Ifakara, Tanzania; 2 School of Biodiversity, One Health and Veterinary Medicine, University of Glasgow, Glasgow, UK; 3 School of Public Health, Faculty of Health Science, University of the Witwatersrand, Johannesburg, South Africa; 4 Wits Research Institute for Malaria, School of Pathology, Faculty of Health Sciences, University of the Witwatersrand, Johannesburg, South Africa; 5 School of Life Science and Biotechnology, Nelson Mandela African Institution of Science and Technology, P. O. Box 447, Arusha, Tanzania

**Correction: Parasites Vectors 17: 38 (2024)** 10.1186/s13071-024-06119-6

Following publication of the original article it was reported that Luca Nelli’s name was misspelled as ‘Luca Neill’.

The original article [1] has been updated.
